# Rectal progesterone administration secures a high ongoing pregnancy rate in a personalized Hormone Replacement Therapy Frozen Embryo Transfer (HRT-FET) protocol: a prospective interventional study

**DOI:** 10.1093/humrep/dead185

**Published:** 2023-09-27

**Authors:** B Alsbjerg, M B Jensen, B B Povlsen, H O Elbaek, R J Laursen, U S Kesmodel, P Humaidan

**Affiliations:** The Fertility Clinic, Skive Regional Hospital, Skive, Denmark; Department of Clinical Medicine, Aarhus University, Aarhus, Denmark; The Fertility Clinic, Skive Regional Hospital, Skive, Denmark; The Fertility Clinic, Skive Regional Hospital, Skive, Denmark; The Fertility Clinic, Skive Regional Hospital, Skive, Denmark; The Fertility Clinic, Skive Regional Hospital, Skive, Denmark; Department of Obstetrics and Gynaecology, Aalborg University Hospital, Aalborg, Denmark; Department of Clinical Medicine, Aalborg University, Aalborg, Denmark; The Fertility Clinic, Skive Regional Hospital, Skive, Denmark; Department of Clinical Medicine, Aarhus University, Aarhus, Denmark

**Keywords:** HRT-FET, low serum progesterone, vaginal progesterone, rectal progesterone, ongoing pregnancy, luteal phase rescue

## Abstract

**STUDY QUESTION:**

Can supplementation with rectal administration of progesterone secure high ongoing pregnancy rates (OPRs) in patients with low serum progesterone (P4) on the day of blastocyst transfer (ET)?

**SUMMARY ANSWER:**

Rectally administered progesterone commencing on the ET day secures high OPRs in patients with serum P4 levels below 35 nmol/l (11 ng/ml).

**WHAT IS KNOWN ALREADY:**

Low serum P4 levels at peri-implantation in Hormone Replacement Therapy Frozen Embryo Transfer (HRT-FET) cycles impact reproductive outcomes negatively. However, studies have shown that patients with low P4 after a standard vaginal progesterone treatment can obtain live birth rates (LBRs) comparable to patients with optimal P4 levels if they receive additionalsubcutaneous progesterone, starting around the day of blastocyst transfer. In contrast, increasing vaginal progesterone supplementation in low serum P4 patients does not increase LBR. Another route of administration rarely used in ART is the rectal route, despite the fact that progesterone is well absorbed and serum P4 levels reach a maximum level after ∼2 h.

**STUDY DESIGN, SIZE, DURATION:**

This prospective interventional study included a cohort of 488 HRT-FET cycles, in which a total of 374 patients had serum P4 levels ≥35 nmol/l (11 ng/ml) at ET, and 114 patients had serum P4 levels <35 nmol/l (11 ng/ml). The study was conducted from January 2020 to November 2022.

**PARTICIPANTS/MATERIALS, SETTING, METHODS:**

Patients underwent HRT-FET in a public Fertility Clinic, and endometrial preparation included oral oestradiol (6 mg/24 h), followed by vaginal micronized progesterone, 400 mg/12 h. Blastocyst transfer and P4 measurements were performed on the sixth day of progesterone administration. In patients with serum P4 <35 nmol/l (11 ng/ml), ‘rescue’ was performed by rectal administration of progesterone (400 mg/12 h) starting that same day. In pregnant patients, rectal administration continued until Week 8 of gestation, and oestradiol and vaginal progesterone treatment continued until Week 10 of gestation.

**MAIN RESULTS AND THE ROLE OF CHANCE:**

Among 488 HRT-FET single blastocyst transfers, the mean age of the patients at oocyte retrieval (OR) was 30.9 ± 4.6 years and the mean BMI at ET 25.1 ± 3.5 kg/m^2^. The mean serum P4 level after vaginal progesterone administration on the day of ET was 48.9 ± 21.0 nmol/l (15.4 ± 6.6 ng/ml), and a total of 23% (114/488) of the patients had a serum P4 level lower than 35 nmol/l (11 ng/ml). The overall, positive hCG rate, clinical pregnancy rate, OPR week 12, and total pregnancy loss rate were 66% (320/488), 54% (265/488), 45% (221/488), and 31% (99/320), respectively. There was no significant difference in either OPR week 12 or total pregnancy loss rate between patients with P4 ≥35 nmol/l (11 ng/ml) and patients with P4 <35 nmol/l, who received rescue in terms of rectally administered progesterone, 45% versus 46%, *P* = 0.77 and 30% versus 34%, *P* = 0.53, respectively. OPR did not differ whether patients had initially low P4 and rectal rescue or were above the P4 cut-off. Logistic regression analysis showed that only age at OR and blastocyst scoring correlated with OPR week 12, independently of other factors like BMI and vitrification day of blastocysts (Day 5 or 6).

**LIMITATIONS, REASONS FOR CAUTION:**

In this study, vaginal micronized progesterone pessaries, a solid pessary with progesterone suspended in vegetable hard fat, were used vaginally as well as rectally. It is unknown whether other vaginal progesterone products, such as capsules, gel, or tablet, could be used rectally with the same rescue effect.

**WIDER IMPLICATIONS OF THE FINDINGS:**

A substantial part of HRT-FET patients receiving vaginal progesterone treatment has lowserum P4. Adding rectally administered progesterone in these patients increases the reproductive outcome. Importantly, rectal progesterone administration is considered convenient, and progesterone pessaries are easy to administer rectally and of low cost.

**STUDY FUNDING/COMPETING INTEREST(S):**

Gedeon Richter Nordic supported the study with an unrestricted grant as well as study medication. B.A. has received unrestricted grant from Gedeon Richter Nordic and Merck and honoraria for lectures from Gedeon Richter, Merck, IBSA and Marckyrl Pharma. P.H. has received honoraria for lectures from Gedeon Richter, Merck, IBSA and U.S.K. has received grant from Gedeon Richter Nordic, IBSA and Merck for studies outside this work and honoraria for teaching from Merck and Thillotts Pharma AB and conference expenses covered by Merck. The other co-authors have no conflict of interest to declare.

**TRIAL REGISTRATION NUMBER (25):**

EudraCT no.: 2019-001539-29

## Introduction

A growing body of scientific evidence supports that reproductive outcome in Hormone Replacement Therapy Frozen Embryo Transfer (HRT-FET) depends on serum progesterone (P4) levels during the mid-luteal phase and early pregnancy. Recently, in a meta-analysis, Melo *etal.* including HRT-FET cohort studies using vaginal progesterone only for luteal phase support (LPS) reported that cycles with P4 levels <32 nmol/l (10 ng/ml) had a significantly lower ongoing pregnancy rate (OPR) and live birth rate (LBR) compared to cycles with higher P4 levels. Furthermore, a significantly higher risk of early pregnancy loss was seen in patients with low luteal P4 levels (Melo *etal.*, 2021). The meta-analysis was based on studies where different cut-off levels of P4 were used. In a previous study, we based the P4 cut-off level on a sensitivity analysis of different P4 levels in relation to OPR. In that study, 35 nmol/l (11 ng/ml) was defined as the best cut-off level and OPR was significantly decreased if P4 was <35 nmol/l (11 ng/ml), OR = 0.54, 95% CI (0.32; 0.91), *P* = 0.022, corresponding to a reduction in chance of an ongoing pregnancy of 14%, CI (−26; −2%), *P *=* *0.024 (Alsbjerg *etal.*, 2018).

The route of progesterone administration impacts serum progesterone levels, and in a meta-analysis by Ranisavljevic *etal.* comparing reproductive outcomes of different progesterone administration routes there were no significant differences between high and low serum progesterone levels. This paradox may be explained by the fact that there are different optimal cut-off levels for different progesterone regimens and other relevant administration routes than the vaginal route (Ranisavljevic *etal.*, 2022).

To improve the reproductive outcome different LPS rescue regimens have been applied. Thus, Cédrin-Durnerin *etal.* doubled the vaginal progesterone dose if P4 was <32 nmol/l (10 ng/ml) measured on the second to fifth progesterone administration day. In their study, a total of 69% of patients reached P4 levels over 32 nmol/l (10 ng/ml) after doubling the vaginal progesterone dose; however, the LBR was significantly reduced compared to the LBR of patients with initially high P4 (17% versus 31%, *P* = 0.02) (Cédrin-Durnerin *etal.*, 2019). Subsequently, four studies added subcutaneous progesterone, 25 mg daily from the day of embryo transfer (ET) as a rescue regimen (Álvarez *etal.*, 2021; Yarali *etal.*, 2021; Labarta *etal.*, 2022; Ozcan *etal.*, 2022). Álvarez *etal.*, Yarali *etal.*, Labarta *etal.*, and Ozcan *etal.* used different cut-off levels 34 nmol/l (10.6 ng/ml), 27.8 nmol/l (8.75 ng/ml), 29 nmol/l (9.2 ng/ml), and 31.8 nmol/l (10 ng/ml), respectively. Interestingly, no significant difference in reproductive outcomes were reported in either of these studies. The LBR’s between the high progesterone group compared to the low progesterone rescue group in two of the studies were, Risk difference = −3.2% (95% CI (−12; 5.7)) and adjusted OR = 0.99 (95% CI (0.79–1.25)) (Álvarez *etal.*, 2021; Labarta *etal.*, 2022). Meaning that additional progesterone administration from the time of blastocyst transfer increased the LBR in patients with an initial low P4 after a standard vaginal progesterone LPS. Interestingly, some studies have suggested that too high mid-luteal P4 levels might decrease reproductive outcomes, in support of a ceiling effect of P4 (Yovich *etal.*, 2015; Alsbjerg *etal.*, 2020; Alyasin *etal.*, 2021).

Progesterone is usually administered vaginally, subcutaneously, orally, or intramuscularly but can also be administered rectally. A few studies used rectally administered progesterone, but until now never as a rescue regimen in HRT-FET cycles (Nillius and Johansson, 1971; Chakmakjian and Zachariah, 1987; Aghsa *etal.*, 2012; Alsbjerg *etal.*, 2020).

The aim of the present study was to investigate the effect on the OPR by administering additional progesterone rectally in HRT-FET cycles in patients with mid-luteal P4 levels lower than 35 nmol/l (11 ng/ml) after a standard vaginal progesterone regimen.

## Materials and methods

### Study design

A prospective interventional study based on serum P4 levels on the day of blastocyst transfer.

### Participants

The study population was recruited at The Fertility Clinic, Skive Regional Hospital, a public fertility clinic in Denmark from January 2020 to November 2022.

The inclusion criteria were: age between 18 and 45 years with at least one autologous vitrified blastocyst for transfer and BMI >18.5 <34 kg/m^2^. Exclusion criteria were an endometrial thickness <7 mm after 12–20 days of 6 mg oestradiol treatment, no blastocyst for transfer after thawing, uterine abnormalities, oocyte donation, and dysregulated severe chronic medical diseases.

**Figure 1.**
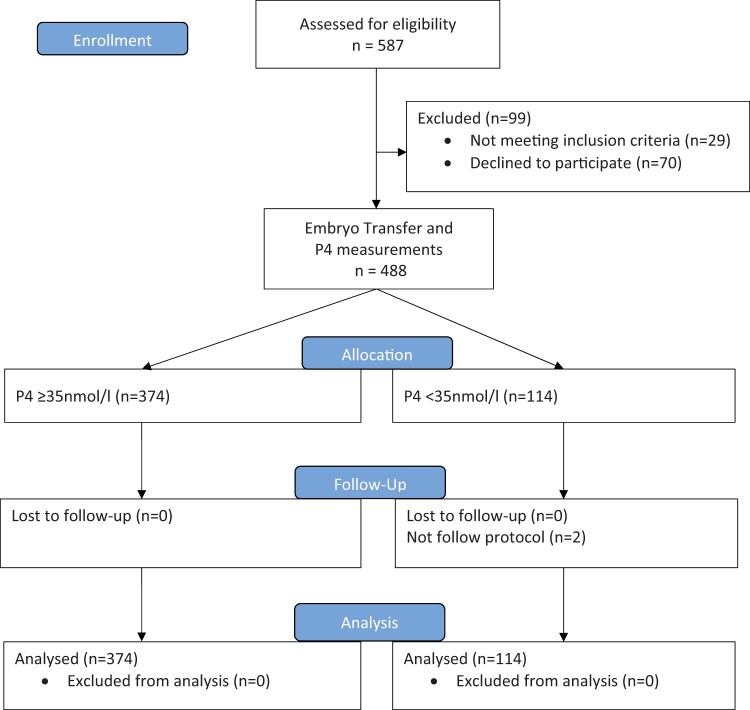
**Study flow diagram.** P4, progesterone.

In total, 488 patients participated in the study, of whom 374 patients had a serum P4 level ≥35 nmol/l (11 ng/ml) at the blastocyst transfer day, and 114 patients had a serum P4 level <35 nmol/l (11 ng/ml) (see study flow chart, Fig. 1).

### Treatment protocols

Endometrial preparation was performed using 6 mg oestradiol valerate daily starting from the second day of the cycle. An ultrasound examination was performed after 12–20 days of treatment and in patients with an endometrial thickness ≥7 mm and quiescent ovaries, treatment with vaginal micronized progesterone 400 mg Cyclogest^®^ (7 am and 7 pm) was initiated. Blastocyst transfer was scheduled for the sixth day of vaginal progesterone treatment, and on this day, serum P4 was measured in a standardized manner 2 h after vaginal progesterone administration. Patients were allocated to one of two groups depending on the serum progesterone levels; <35 nmol/l (11 ng/ml) or ≥35 nmol/l (11 ng/ml).

If serum P4 was <35 nmol/l (11 ng/ml) (study group) the vaginal progesterone regimen was supplemented with additional 400 mg Cyclogest^®^ bid (7 am and 7 pm) administered rectally starting on the evening of the day of transfer. In case of a positive pregnancy test patients continued their rectal rescue LPS regimen; 2 vaginal + 2 rectal suppositories (2 + 2) until gestational week 8 + 0. If the ultrasound examination visualized an intrauterineviable pregnancy, the vaginal progesterone administration continued alongside 6 mg oestradiol valerate until gestational week 10 + 0.

If serum P4 was ≥35 nmol/l (11 ng/ml) on the day of blastocyst transfer, the standard LPS regimen continued, and in pregnant patients, all LPS was discontinued at gestational week 10 + 0. Pregnancy scan was performed in gestational weeks 7–8 and week 12 for all pregnant women.

An ongoing pregnancy was defined as a viable pregnancy at the ultrasound scan performed at gestational week 12.

**Figure 2.**
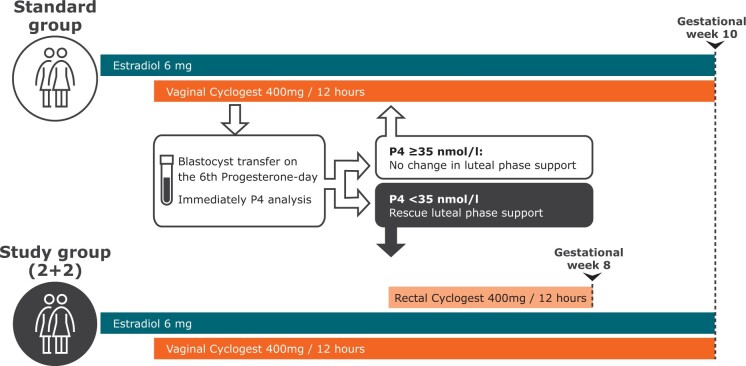
**Study protocol.** P4, progesterone. Note timings are not to scale.

Study flow diagram are shown in Fig. 2.

### Embryos and embryo transfer

Double embryo transfer (DET) was permitted according to the protocol; however, only two DET were performed. Four hundred and eighty-six were single embryo transfer (SET) and all blastocyst transfers were autologous blastocysts vitrified on Day 5 or 6, using the ‘Cryotec method’ by Masashige Kuwayama (Gandhi *etal.*, 2017). Blastocysts were scored according to the Gardner and Schoolcraft grading system (Gardner and Schoolcraft, 1999) and blastocyst transfer was scheduled for the sixth day of vaginal progesterone administration. A top-quality blastocyst (Score 1) was defined as a 3AA, 3AB, 3BA, 4AA, 4AB, 4BA, 5AA, 5AB, and 5BA. An intermediate blastocyst (Score 2) was defined as a 3BB, 4BB, and 5BB and no poor-quality blastocysts (Score 3) were transferred.

### Blood sampling and hormone analyses

Blood sampling was carried out in a standardized way on theblastocyst transfer day (9 am to 11 am) 2–4 h after vaginal progesteroneadministration. Pregnancy testing was performed 9–11 days after ET at a random time for patient convenience and serum P4 was included in the analysis. Serum P4 levels were analysed using direct chemiluminescent technology (Atellica, Siemens), routinely used for analysis at the local department of biochemistry. All measurements were performed according tothe manufacturer’s instructions. The assay provided resultsfrom 1.0 to 1908 nmol/l and was designed to have a within-laboratory precision of ±12% (2 Coefficient of Variation) for samples level 6.3 nmol/l and ±8% (2 CV) at samples level 39 nmol/l.

All blood samples were analysed for progesterone immediately.

### Sample size calculation

The power calculation was based on previous studies reporting that about half of patients had serum P4 levels <35 nmol/l following two different standard vaginal LPS regimens (Labarta *etal.*, 2017; Alsbjerg *etal.*, 2018). Furthermore, we previously reported an OPR of 51% in the ≥35 nmol/l group and 38% in the <35 nmol/l group if no additional progesterone was administered in HRT-FET (Alsbjerg *etal.*, 2018).

Based on the assumption that progesterone supplementation in the low P4-group would increase from 38% to 51%, a Power (1 − β) of 80%, α = 5%, one-sided test, a sample of 112 participants would be required in each of the groups.

The one-side test was chosen given that increasing the progesterone dose by rectal administration would increase the serum level, and hence the pregnancy rate would either remain the same or increase. Furthermore, increasing the serum progesterone from ET day has shown to be beneficial (Álvarez *etal.*, 2021; Yarali *etal.*, 2021; Labarta *etal.*, 2022).

In total, 224 patients were needed with 112 in the low P4 group (<35 nmol/l). Two patients with P4 <35 nmol/l (11 ng/ml) violated the protocol; one did not administer rectally progesterone at all and the other did not administer rectally progesterone for 1 week (gestational week 5). Consequently, the total number of patients with P4 <35 nmol/l was increased to 114. All 488 patients enrolled were included in the final analysis.

The following assumptions were made: very limited loss to follow-up, near full compliance to study medication, and homogeneity in the treatment effect.

### Statistical methods

Normality was evaluated using quantile-quantile plots, and the assumption of equal variance was tested using the F-test. Fisher’s exact, chi-squared, and *t*-test were used as appropriate. Furthermore, a logistic regression model was used to adjust for potential confounders.

All statistical analyses were performed using STATA version 13, StataCorp LLC, USA.

### Ethics

Approval by the Regional Ethical Committee, the Danish Data Protection and the Danish Medicines Agency was given on 10 October 2019. The study was registered in EudraCT no.: 2019-001539-29. Furthermore, the trial was monitored by the Good Clinical Practice (GCP) unit at Aarhus University with GCP protocol number: 787/2019.

All participating patients signed a letter of consent before enrolment in the study and no patients withdrew consent during the study.

## Results

### Patients characteristics

Among the 488 HRT-FET cycles, the mean age of the patients at oocyte retrieval (OR) was 30.9 ± 4.6 years and the mean BMI at ET 25.1 ± 3.5 kg/m^2^. The mean BMI in the rescue group (2 + 2) was 24.8 ± 3.5 versus 26.0 ± 3.4 kg/m^2^ in the standard group, but this difference was non-significant.

**Figure 3.**
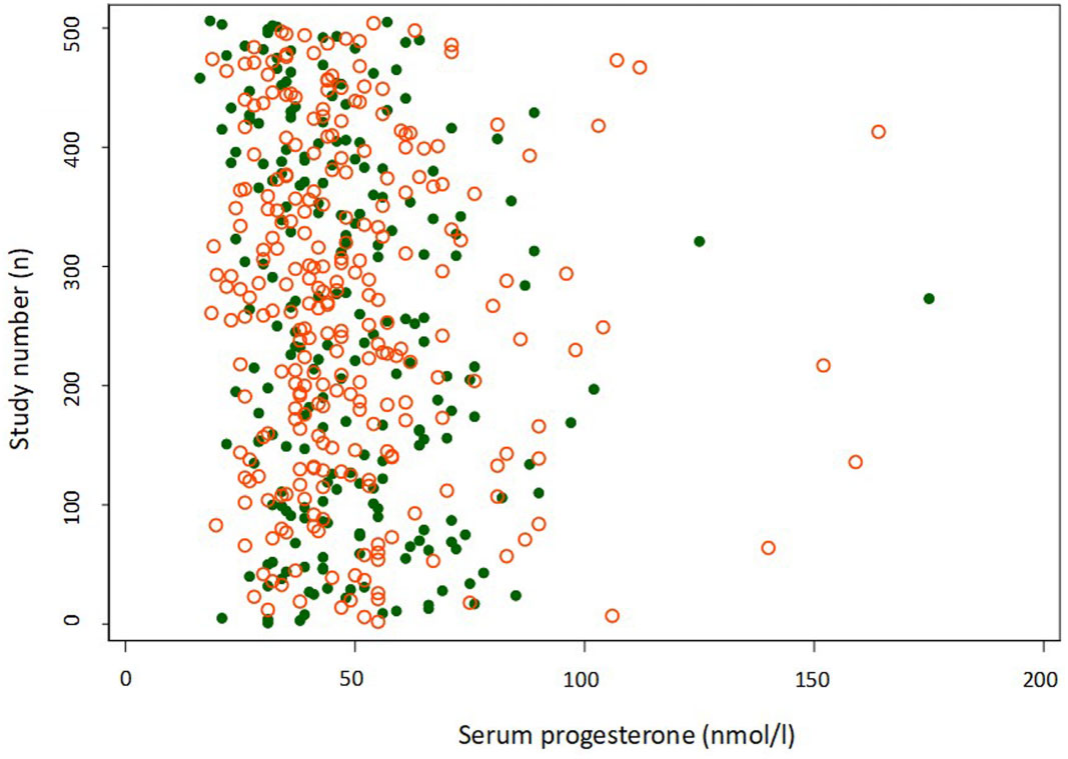
**Plot of serum progesterone levels at blastocyst transfer day.** Green dot ongoing pregnancy; red ring no ongoing pregnancy.

The mean serum P4 level on the day of blastocyst transfer was48.9 ± 21.0 nmol/l (15.4 ± 6.6 ng/ml) and 51.6 ± 26.3 nmol/l (16.2 ± 8.3 ng/ml) on the pregnancy test day. A total of 23% (114/488) of the patients had a serum P4 level lower than 35 nmol/l (11 ng/ml). The range of P4 levels was 16 to 175 nmol/l (5.0 ng/ml to 55.0 ng/ml) and the 10th percentile was 28 nmol/l (8.8 ng/ml). Furthermore, the 90th percentile of P4 levels on the ET day was 73 nmol/l (23.0 ng/ml), and only 12 patients (2%) had P4 levels >100 nmol/l (31.4 ng/ml). The distribution of serum P4 levels in relation to having an ongoing pregnancy or not is shown in Fig. 3. SET was performed in 486 cycles (99.6%). Significantly more anovulation/oligoovulation patients were seen in the 2 + 2 group; 21% (24/114) compared to 9% (35/374) in the standard group (*P* = 0.001). For further characteristics see Table 1.

**Table 1.** Basic characteristics of patients.

	All	Standard	2 + 2*	*P*-value
		P4 ≥35 nmol/l	P4 <35 nmol/l	
Cycles, n	488	374	114	
Serum P4 ET-day, mean (SD)	48.9 (21.0)	55.1 (20.0)	28.3 (4.4)	<0.01
Serum P4 pregnancy test day, mean (SD)	51.6 (26.3)	46.4 (19.5)	69.2 (36.7)	<0.01
Age OR, years, mean (SD)	30.9 (4.6)	30.9 (4.3)	30.9 (4.6)	0.46
Age ET, years, mean (SD)	31.5 (4.4)	31.4 (4.4)	31.6 (4.7)	0.39
Body mass index, kg/m^2^, mean (SD)	25.1 (3.5)	24.8 (3.5)	26.0 (3.4)	0.65
Smoking	6 (1.2)	4 (1.0)	2 (1.8)	0.56
Single embryo transfer	486 (99.6)	372 (99.5)	114 (100)	0.43
Developmental stage				0.82
Day 5 blastocyst transfer	425 (86.9)	325 (86.6)	100 (87.7)	
Day 6 blastocyst transfer	63 (12.9)	49 (13.1)	14 (12.3)	
Insemination method				0.81
IVF	222 (45.5)	169 (45.2)	53 (46.5)	
ICSI	266 (54.5)	205 (54.8)	61 (53.5)	
No. of cycles with at least one high quality blastocyst^1^	345 (70.7)	263 (70.3)	82 (71.9)	0.71
Primary diagnosis				<0.01
Anovulation	59 (12)	35 (9)	24 (21)	
Idiopathic	133 (27)	103 (28)	30 (26)	
Male factor	186 (38)	146 (39)	40 (35)	
Tubal factor	34 (7)	32 (9)	2 (2)	
Single, n (%)	54 (11)	42 (11)	12 (11)	
Others,^2^ n (%)	22 (5)	16 (4)	6 (5)	

Values are n (%) unless stated otherwise.

* 2 + 2: luteal phase rescue regime including two vaginally and two rectally administrations from blastocyst transfer day.

P4, progesterone; ET, embryo transfer; OR, oocyte retrieval.

1 High-quality blastocyst was defined as 3AA, 3AB, 3BA, 4AA, 4AB, 4BA, 5AA, 5AB, and 5BA (Gardner classification).

2 Others = include endometriosis, recurrent pregnancy loss, and ovarian causes.

No adverse events were reported during the study.

### Reproductive outcomes

The overall, positive hCG rate, clinical pregnancy rate, OPR week 12, and total pregnancy loss rate were 66% (320/488), 54% (265/488), 45% (221/488), and 31% (99/320), respectively.

There was no significant difference in OPR week 12 between the standard group and the 2 + 2 group 45% (168/374) versus 46% (53/114) *P* = 0.77, respectively. Neither were differences seen as regards total pregnancy loss 30% versus 34% (*P* = 0.53) nor biochemical pregnancy loss 18% versus 16% (*P* = 0.73), Table 2.

**Table 2.** Reproductive outcome.

	All	Standard	2 + 2*	*P*-value
	(488)	P4 >35 nmol/l	P4 <35 nmol/l	
		(374)	(114)	
Pregnancy per ET	320 (66)	240 (64)	80 (70)	0.29
Clinical pregnancy per ET	265 (54)	198 (53)	67 (59)	0.27
Ongoing week 12 per ET	221 (45)	168 (45)	53 (46)	0.77
Ongoing gemelli week 12	1	1	0	
Total pregnancy loss	99 (31)	72 (30)	27 (34)	0.53
Biochemical pregnancy loss	56 (18)	43 (18)	13 (16)	0.73

Values are n (%).

* 2 + 2: luteal phase rescue regime including two vaginally and two rectally administrations from blastocyst transfer day.

P4, progesterone; ET, blastocyst transfer.

A logistic regression analysis adjusting for BMI, age at OR, day of vitrification, and blastocyst score showed that OPR did not depend on whether patients had initially low P4 and were rescued with rectal progesterone or were above the P4 cut-off, OR 1.06 (95% CI (0.68–1.64), *P* = 0.80). Age at OR and blastocyst score correlatedwith OPR week 12, OR 0.95 (95% CI (0.91–0.99) *P* = 0.03) and OR 0.51 (95% CI (0.33–0.77), *P* < 0.01) (see Table 3).

**Table 3.** Logistic regression analysis of the association between group and chance of an ongoing pregnancy week 12, adjusted for body mass index, age at oocyte retrieval (OR), day of vitrification, and blastocyst score.

Characteristics	Odds ratio	95% CI	*P*-value
Group			
Standard group	1		
2 + 2 group	1.06	0.68–1.64	0.80
Body mass index	0.98	0.93–1.04	0.58
Age at OR, years	0.95	0.91–0.99	0.03
Day of vitrification			
5	1		
6	0.60	0.33–1.11	0.10
Blastocyst score, quality			
High	1		
Medium	0.51	0.33–0.77	<0.01

OR, oocyte retrieval.

## Discussion

This prospective interventional study in HRT-FET shows that additional rectal administration of progesterone in patients with P4 ˂35 nmol/l after a standard vaginal administration rescued the luteal phase and resulted in a non-significant difference in OPR week 12 in patients below and above the cut-off level of ≥35 nmol/l (11 ng/ml).

It has become evident that a substantial part of all HRT-FET cycles treated with a standard vaginal progesterone end up withan insufficient luteal phase when evaluated by serum P4 measurement (Melo *etal.*, 2021); even though, serum P4 must be regarded as a proxy marker of the endometrial micro-environment (Labarta *etal.*, 2021).

In the present study, 35 nmol/l (11 ng/ml) was chosen as the optimal P4 cut-off level. This was based on the results of a former study from our group (Alsbjerg *etal.*, 2018), in which the cut-off was defined by a sensitivity analysis, comparing OPRs and different serum P4 levels in a cohort similar to the present study cohort. Others used different cut-off levels based on the lowest quartile or the 30th percentile (Labarta *etal.*, 2017, 2020) equal to 29.3 nmol/l (9.2 ng/ml) and 28.0 nmol/l (8.8 ng/ml), respectively, and Gaggiotti-Marre *etal.* defined their reported optimal cut-off using the median [IQR] which was 33.8 [25.7; 41.8] nmol/l (10.64 (8.08; 13.13) ng/ml) (Gaggiotti-Marre *etal.*, 2018). We must keep in mind that serum P4 levels are influenced by the vaginal progesterone product used (ESHRE abstract, p-595, 2023) and particularly the population, as serum levels are impacted by BMI, age, smoking, ethnicity, and, importantly, the timing of the blood sampling (González-Foruria *etal.*, 2020; Maignien *etal.*, 2022). Furthermore, the 35 nmol/l (11 ng/ml) cut-off level is in accordancewith a recent meta-analysis showing a significantly higher LBR/OPR using cut-off levels between 32 and 64 nmol/l (10–20 ng/ml) (Melo *etal.*, 2021); moreover, 35 nmol/l (11.0 ng/ml) was also the cut-off found by Labarta *etal.* (2017) when both sensitivity and specificity for OPR were >50% (70.0% sensitivity, 50.5% specificity). If a 32 nmol/l (10 ng/ml) P4 cut-off level was used in the present study, only 82 patients would be categorized as low P4 and not 114 patients.

In the present study, the rectally administered progesterone was terminated at gestational week 8 + 0. The rationale for this is the substantial contribution of progesterone from the placenta at this time point. The placental secretion of progesterone in earlypregnancy was evaluated by Kawachiya *etal.* in a cohort of HRT-FET cycles resulting in a live birth, and the progesterone used in the HRT-FET cycles included exclusively synthetic progesterone. The authors reported medians and interquartile ranges [IQRs] of natural progesterone in gestational week 7 + 5 and 8 + 3 to be 23.5 [15.0; 31.2] nmol/l (7.4 (4.7; 9.8) ng/ml) and 34.7 [22.9; 42.6] nmol/l (10.9 (7.2; 13.4) ng/ml), respectively (Kawachiya *etal.*, 2019). The data illustrate that a substantial part of women in gestational week 8 will have a contribution from the placenta which equals the contribution from the exogenous progesterone administered in a standard vaginal progesterone regimen.

The 2 + 2 rescue regimen used in the present study showed no significant difference in OPR between patients with P4 levels above and below 35 nmol/l at blastocyst transfer day when the rescue regimen was applied for the low P4 group. Our results areconsistent with the results reported by Álvarez *etal.* (2021) and Labarta *etal.* (2022), using 25 mg progesterone SC daily as a rescue regimen in patients with P4 levels lower than 33.7 nmol/l (10.6 ng/ml) and 29.3 nmol/l (9.2 ng/ml); respectively. The percentageof patients needing recue was 39% and 25% in thosestudies in which both the dosing of micronized vaginal progesterone (200 mg/8 h vs 400 mg/12 h) and the cut-off levels were different (Álvarez *etal.*, 2021; Labarta *etal.*, 2022). In the present study, only 23% of patients needed LPS rescue, and this may be explained by another vaginal progesterone product used in our cohort. The mean P4 level was 48.9 ± 21.0 nmol/l (15.4 ± 6.6 ng/ml) and, thus higher compared to a mean P4 level of 41.0 ± 22.0 nmol/l (12.9 ± 6.9 ng/ml) and 41.0 ± 28.6 nmol/l (12.9 ± 9.0 ng/ml) in the Álvarez *etal.* (2021) and Labarta *etal.* (2022) cohorts; furthermore, in the present study a very strict time frame of blood sampling was applied.

Other rescue regimens including oral and intramuscular (IM) progesterone administration have also been reported to have a good rescue effect (Stavridis *etal.*, 2023). However, the potential advantages of the 2 + 2 rescue regimen are that patients already have the medication at home, the cost may be lower compared to SC and IM progesterone, and the fact that the majority of patients found the rectal route to be more acceptable and with fewer side-effects in terms of discharge compared to the vaginal route (ESHRE 2023 abstract p-555).

### Pregnancy loss after HRT

The risk of pregnancy loss is decreased in HRT-FET cycles if serum P4 is optimal. Based on a meta-analysis Melo *etal.* reported a risk ratio of 0.62 (95% CI (0.50–0.77)) for miscarriage if serum P4 was higher than 32 nmol/l (10 ng/ml). Interestingly, a previous study highlighted that the risk of miscarriage was higher in HRT-FET cycles compared to the true natural cycle (t-NC) with LPS and the modified natural cycle (m-NC) triggered with HCG; the analysis included a total of 4474 FET cycles and the total pregnancy loss rates were 41.5%, 22.4% and 33.6% (*P* < 0.0001), for HRT-FET, true natural cycle and modified natural cycle, respectively (Tomas *etal.*, 2012). Ten years ago, luteal phase serum P4 levels were not routinely measured which might explain the higher pregnancy loss as the standard LPS used in the Tomas *etal.* cohort was vaginal micronized progesterone 90 mg twice daily which we subsequently learned results in a mean serum P4level of 24.2 ± 10.1 nmol/l (7.6 ± 3.2 ng/ml) (Alsbjerg *etal.*, 2021). In the present study, no significant difference was seen in totalpregnancy loss rate between the patient group with P4 ≥ 35 nmol/l (11 ng/ml) and the low P4 rescue group 30% vs 34% (*P* = 0.53), respectively.

Whether a further decrease in pregnancy loss rate is achievable in a cohort of un-screened blastocysts is unknown. As In comparison Gaggiotti-Marre *etal.* found an overall miscarriage rate of 13.5% in a cohort of tNC FET with no LPS; however, in that study, 29% of the blastocyst were euploid (PTG-A screened) (Gaggiotti-Marre *etal.*, 2018).

### Is there a risk of too high luteal phase P4 levels in HRT-FET?

A few studies previously suggested that too high mid-luteal P4 levels in HRT-FET negatively impacted the reproductive outcome. Thus, Yovich *etal.* reported that, although not significant, patients with P4 levels higher than 100 nmol/l (31.4 ng/ml) treated with progesterone 400 mg/8 h had a lower LBR compared to patients with P4 between 70 and 100 nmol/l (22–31 ng/ml) (Yovich *etal.*, 2015).

The standard vaginal progesterone regimen used in the present HRT-FET protocol ensured that 77% of patients had a P4 level >35 nmol/l (11 ng/ml), and only 12 patients (2%) had P4 levels >100 nmol/l on the day of ET (31.4 ng/ml). Interestingly, 9 out of the 12 patients did not achieve an OPR week 12.

### Parameters influencing P4 levels

Cédrin-Durnerin *etal.* in a retrospective analysis showed that doubling the vaginal progesterone dose in patients with P4 <10 ng/ml (31.8 nmol/l) on the ET day did not increase LBRs. Furthermore, the mean serum progesterone in the low P4 group was the same before and after increasing the vaginal progesterone dose (Cédrin-Durnerin *etal.*, 2019). Thus, it might be hypothesized that after a certain progesterone dose, the maximal vaginal absorption capacity is reached and that additional administration will not increase P4 levels further. However, this vaginal absorption capacity might be different from patient to patient and importantly, will differ between vaginal products.

Serum P4 levels fluctuate in relation to time of administration and consequently blood sampling time in the present cohort was strictly standardized from 2 to 4 h after the administration of vaginal progesterone. The P4 level during this time frame for most patients reflects the highest P4 level (Duijkers *etal.*, 2018). However, it has never been studied whether this is the best prognostic serum P4 value as regards to reproductive outcomes, compared to the P4 level just before administration which mirrors the lowest P4 level.

It has been shown that BMI, age, a history of low P4, and the time of blood sampling in relation to the administration influence the P4 levels (González-Foruria *etal.*, 2020). In another cohort reported by Maignien *etal.* parity and a non-European geographic origin were also associated with low P4 levels. Furthermore, active smoking appears to be associated with higher P4 levels which may be explained by decreased metabolic clearance of steroid hormones (Maignien *etal.*, 2022).

In the present cohort, significantly, more patients were anovulatory in the 2 + 2 group compared to the standard group (21% vs 9%).

### Power calculation

The total number enrolled in this study was 488 patients, although,it was originally powered to 224 patients of which 112would have P4 <35 nmol/l (11 ng/ml). The explanation is that the power calculation was based on another standard vaginal progesterone regimen in which 50% of patients had P4 levels<35 nmol/l (11 ng/ml) (Alsbjerg *etal.*, 2018). The vaginal progesterone product used in the present study (Cyclogest^®^ 400 mg/12 hours) resulted in a mean serum P4 of 48.9 ± 21.0 nmol/l (15.4 ± 6.6 ng/ml) and only 23% of patients had P4 levels<35 nmol/l (11 ng/ml). Consequently, more patients were enrolled to include enough patients to the rescue group.

### Limitations

Formulations of vaginal micronized progesterone products differ, as progesterone can be suspended in vegetable hard fat, in a vegetable lipophile liquor, are prepared in an oil-in-water emulsion carrier, or mixed in a tablet. The product used in this study was suspended in vegetable hard fat, and it is unknown whether other products would have the same effect when administered rectally.

Due to national clinical guidelines recommending weight restriction for IVF patients no patients with BMI more than 34 kg/m^2^ were included; consequently, it is unknown whether patients with higher BMI will benefit from the rectal rescue regimen.

Seventy patients declined to participate in the study, which is 12% of all patients given the first information regarding the study. No patient refused to participate due to the possibility of needing rectal administration. Regarding patient convenience, more patients in the rescue group would prefer rectal administration over vaginal administration if they were to choose theprogesterone administration route themselves (ESHRE 2023, abstract p-555).

### Future research

There are still a number of unanswered questions regarding the most optimal HRT-FET protocol, and individualization of the luteal phase support has only just begun. Sub-groups of patients may need higher luteal P4 levels for successful implantation as recently shown for the endometriosis/adenomyosis patient (Alsbjerg *etal.*, 2023). Furthermore, it needs to be clarified at which time point P4 should be measured to obtain the best predictivevalue. Vaginal progesterone products may also need to be re-evaluated to ensure the best dosing regimen for each product.

## Conclusion

A substantial part of HRT-FET patients receiving vaginal progesterone treatment has low luteal serum P4. Rectal rescue in these patients secures the reproductive outcome.

## Data Availability

The data underlying this article will be shared upon reasonable request to the corresponding author.
